# Assessment of the coordination of low-carbon development and socio-economic development based on a comprehensive model: A case study of Anhui Province (China)

**DOI:** 10.3389/fpubh.2022.884342

**Published:** 2022-07-22

**Authors:** Qinfeng Xing, Ziwei Yang

**Affiliations:** State Key Laboratory of Mining Response and Disaster Prevention and Control in Deep Coal Mines, Anhui University of Science and Technology, Huainan, China

**Keywords:** low-carbon development, entropy weight method, coupling coordination level model, synergy degree model, grey forecasting method, Anhui Province (China)

## Abstract

The coordination of low-carbon development and socio-economic development has been the favorite subject of carbon emission reduction with the proposal of “dual carbon” goal. So an evaluation indicator system based on four dimensions of resource environmental endowment, energy environmental endowment, economic development endowment and social development endowment was constructed. And then, entropy weight method, coupling coordination level model, synergy degree model and gray forecasting method were used for comprehensive analysis based on the data from 2011 to 2020 in Anhui Province (China). The results show: 1) Low-carbon development lagged behind socio-economic development; 2) Low-carbon development and socio-economic were mutually beneficial; 3) The coordinated level of low-carbon development and socio-economic development in Anhui province was continuously improved, and the ordered structure will be realized in 2024 and 2029 respectively. This study deepens the theoretical cognition of carbon emission reduction, and the following countermeasures are formed: Highlighting the strategic guidance of sustainable development, perfecting the new regulatory mechanisms for carbon emission reduction, and innovating the science and technology to resolve major development crises.

## Introduction

The global greenhouse effect is becoming more and more serious, which has a weaker risk of impact on biodiversity, ecosystems and their functions. In the face of increasingly severe global climate change situation, the concept and model of low-carbon economy emerged at the historic moment. The concept of low-carbon economy first appeared in the Energy White Paper issued by the UK in 2003 (1). In 2006, Nicholas Stern, a famous economist, pointed that “low-carbon economy” is an emerging economic form that includes low-carbon industries and low-carbon technologies. Therefore, low-carbon economy is an economic development model based on low energy consumption, low pollution and low emissions, which is mainly manifested in the improvement of energy efficiency, optimization of energy structure and rationalization of consumption behavior (2, 3). Furthermore, in order to cultivate people's awareness of low-carbon consumption, the United States has attached “carbon labels” on many products to promote the positive development model of carbon emission reduction (4). In order to support low-carbon economy, the EU actively promoted the “Green Economic Recovery” plan (5). In the case of low-carbon economy in China, at the Copenhagen climate conference, the Government pledged to cut CO2 emissions per unit of gross domestic produc by 40–45% by 2020 compared with 2005 levels. In the CHINA-US Joint Statement on Climate Change issued by China and the US in 2014, China officially stated for the first time that China's carbon emissions are expected to peak around 2030, and that the proportion of non-fossil energy in primary energy will increase to 20% by 2030.

Moreover, low-carbon development is an important part of low-carbon economy, which is a kind of development patterns based on clean and efficient use of energy, characterized by low carbon emissions and low environment pollution, which mainly refers to the decline in CO2 emissions per unit of gross domestic product as the economy develops (6, 7). More than that, low-carbon development also means the minimization of carbon emissions under the condition of ensuring the sustainable socio-economic development (8). However, most of the current studies on low-carbon development focus on the fields of industry to explore the path of low-carbon development [(9–12)]. Research on the broader scope of low-carbon development and its interaction with socio-economic development is scarce, which is also essential for low-carbon development [(13, 14)]. Additionally, according to the low-carbon development characteristics in China, the coupling level of low-carbon development and socio-economic development is full of important practical significance, which is more in line with the reality of China's socio-economic development and can further promote low-carbon development. This is because that coordination means links, and its essence is a balancing act. It is the global or collective effect produced by the interaction of a large number of subsystems in an open system. It can make the system change from disorder to order at the critical point and produce some stable structure from chaos.

According to the existing literature, coupling coordination level model is used to describe the correlation and interaction mechanism among a number of study objects, such as the coupling level of urbanization and land use carbon emissions [(15, 16)]; the coupling level of economic development and ecological environment protection (17); the coupling level among carbon emissions - industrial structure - regional innovation (18). Furthermore, synergy degree model also is used to describe the mechanism of action between economy and ecology, such as the synergy effect of low-carbon economic development (19); the synergy of green technology innovation and low-carbon development (20); synergistic evaluation of the scientific and technological innovation and the ecological environment (21). Additionally, considering these opinions, there are similarities and differences between the two models, but they are always used independently in previous studies. However, the two analysis models used together can better avoid the uncertainty during the coordination process and accurately identify the time series changes of synergy degree. Therefore, the above two models are used in the paper, so as to enrich the corresponding study system.

Additionally, based on the theory and practice research of low-carbon development, this paper analyzes the coupling level and the synergy degree of low-carbon development and socio-economic development *via* a comprehensive model based on the data 2011 to 2020 in Anhui province (China), and then some reference are provided to help the early realization of dual carbon strategy.

## Materials and Methods

### Study area

**Figure 1:**
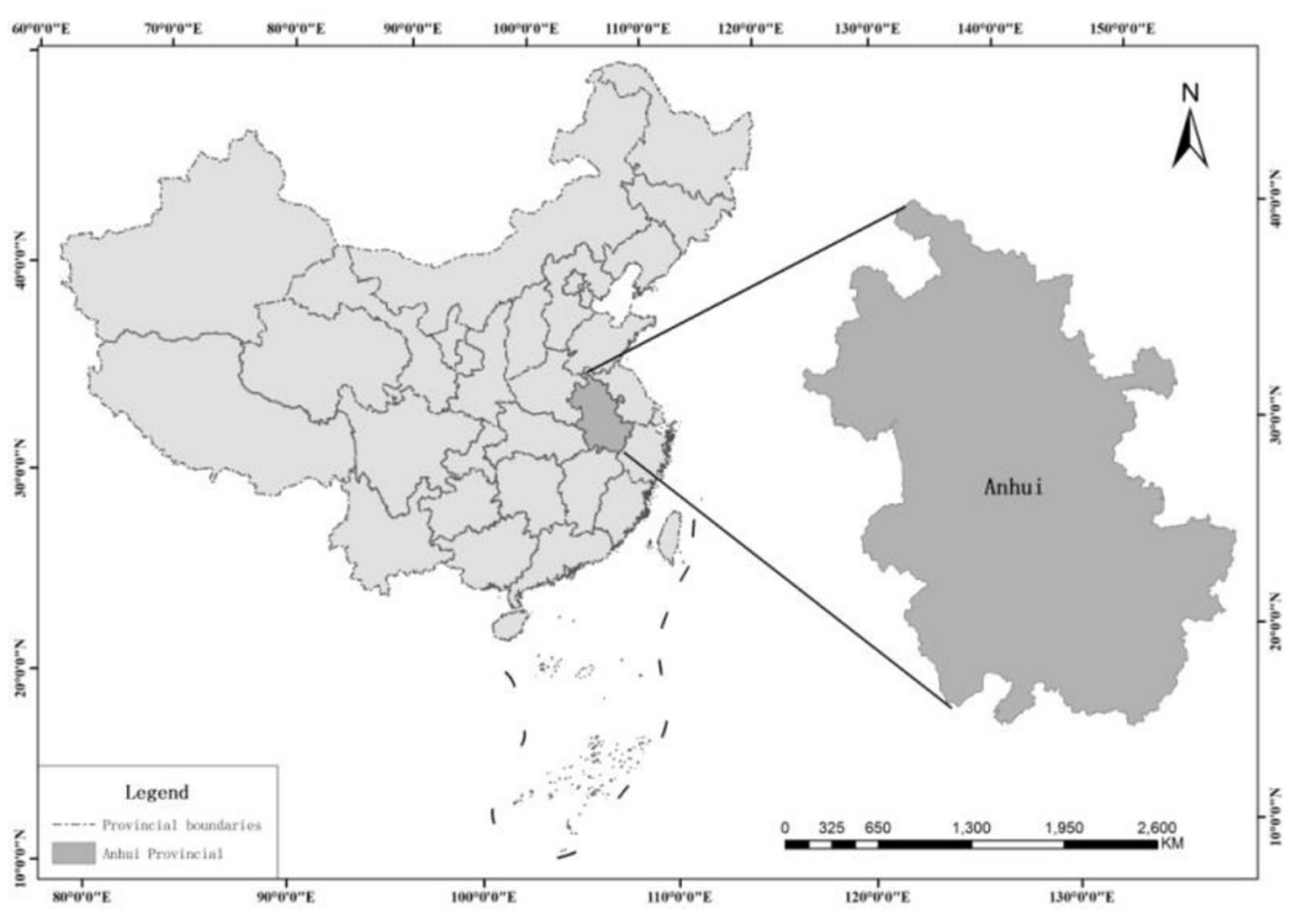
Location of Anhui province in China.

Anhui province, is located in east China, and in the middle and lower reaches of the Yangtze River and Huaihe River, and the hinterland of the Yangtze River Delta (Figure 1), between 114 ° 54′-119 ° 37′ E and 29 ° 41′-34 ° 38′ N. It belongs to the central and eastern economic zones, namely, the Yangtze River Delta urban agglomeration. The urban agglomeration is formed together with Jiangsu Province, Zhejiang Province and Shanghai City, which has become one of the six world-class urban agglomerations in the world (22). During the period of the 13th five-year plan, the economy maintained rapid growth, and the development pattern was upgraded from “Medium in total amount and low in per capita” to “Medium in total amount and low in per capita”. Although the economic structure was perfected, and its low-carbon development continued to push forward, but the coordination of low-carbon development and socio-economic development in Anhui province is still not very satisfactory. Furthermore, as an energy province in the central region (China) and an agricultural province that relies on economies of scale, carbon emission reduction is still the main shackle to achieve low-carbon development and dual carbon strategy. Therefore, how to avoid the uncertainty during the coupling level of low-carbon development and socio-economic development in Anhui province and accurately identify its synergy degree is of great practical significance.

### Indicator construction

To achieve the expected results of coordinated and synergistic effects of low-carbon development and socio-economic development, as well as the principles of availability, scientificity and objectivity are upheld, representative indicators are selected in the fields of them, and their evaluation system is designed in Table 1.

**Table 1:** Comprehensive indicator evaluation system.

**Target layer**	**Criterion layer**	**Indicator layer**	**Attribute**
Low-carbon system development level	Resource environmental endowment	Forest Coverage (X1)	+
		Air Quality Index excellent rate (X2)	+
		Resource yield rate (X3)	+
	Energy environment endowment	Coal production capacity (X4)	−
		Coal consumption (X5)	−
		Per unit of GDP energy consumption (X6)	−
Socio-economic system development level	Economic development endowment	Per disposable income (X7)	+
		Tertiary sector of the economy of GDP (X8)	+
		Strategic emerging industries output value (X9)	+
	Social development endowment	Urbanization rate (X10)	+
		Minimum subsistence allowance for urban residents (X11)	−
		Number of graduate students in school (X12)	+

### Entropy weight method

Entropy weight method was used to deal with the indicator system of low-carbon development and socio-economic development.

#### Standardized treatment

In order to eliminate the dimensional difference, the positive and negative indicators were standardized by the extreme value method (shown in Eq. Eq. 1 and Eq. 2).


(1)   Positive index: yij = xij-min xijmax xij-min xij×0.999+0.001

(2) Negative index: yij = max xij-xijmax xij-min xij×0.999+0.001

#### Determine the index weight

The information entropy *ej* and the weight of each index ω_*j*_ were obtained by Eq. 3, Eq. 4 and Eq. 5.


(3) $$\begin{array}{l} ej\;=\;-\frac{1}{\ln\;m}\overset{m}{\underset{i=1}{\sum }}fij\ln\;fij \end{array}$$

(4) $$\begin{array}{l} fij\;=\;\frac{yij+1}{\overset{m}{\underset{i=1}{\sum }}\left(yij+1\right)} \end{array}$$

(5) $$\begin{array}{l} \omega_{j}\;=\;\frac{1-ej}{n-\overset{n}{\underset{j=1}{\sum }}ej} \end{array}$$

#### Comprehensive development index

Comprehensive development index represents the comprehensive level of low-carbon development and socio-economic development, and its indicator value has a positive correlation with their development level. The concrete calculation formula is obtained by Eq. 6, Eq. 7, and Eq. 8.


(6) $$\begin{array}{l} F\left(x\right)\;=\;\overset{n_{1}}{\underset{j=1}{\sum }}a_{j}\times X_{j} \end{array}$$

(7) $$\begin{array}{l} G\left(y\right)\;=\;\overset{n_{2}}{\underset{j=1}{\sum }}b_{j}\times Y_{j} \end{array}$$

(8) $$\begin{array}{l} T\;=\;\alpha F\left(x\right)+\beta G\left(y\right) \end{array}$$

In the formula: *F*(*x*) and *G*(*y*) represent low-carbon development index and socio-economic development index respectively; *a*_*j*_ and *b*_*j*_ represent the weight of the indicator *j* in low-carbon development and socio-economic development respectively; *X*_*j*_ and *Y*_*j*_ are standardized values of indicator *j* in low-carbon development and socio-economic development respectively; *n*_1_ and *n*_2_ are the number of indicators of low-carbon development and socio-economic development respectively. Through the results study of numerous scholars (23–25), this paper presumes that low-carbon development is as important as socio-economic development, so α = β = 0.5; *T* is a comprehensive development index.

### Coupling coordination level model

#### Coupling level

The *C* on the interaction between low-carbon development and socio-economic development can be coupled by Eq. 9.


(9) $$\begin{array}{l} C\;=\;{\left(\frac{2F\left(x\right)G\left(y\right)}{F\left(x\right)+G\left(y\right)}\right)}^{k} \end{array}$$

Where, *C* is the coupling level, which reflects the coordination level between low-carbon development and socio-economic development, and the larger the value of *C*(0 ≤ *C* ≤ 1), the better the coupling between the two systems, and vice versa. ^*k*^ is the adjustment factor, generally 2 ≤ *k* ≤ 5, in order to increase the level of differentiation, this study takes *k* = 3.

#### Coupling coordination level

Compared with the coupling level model, the coupling coordination level model has higher stability and wider scope of application, and the former can't judge the defect of benign and malignant coupling. The study object can be extended to the same region or different time, and quantitative evaluation and comparison, with a strong operability (shown in Eq. 10).


(10) $$\begin{array}{l} D\;=\;\sqrt{C\;\times \;T} \end{array}$$

Where, *D* is the level of coupling coordination.

#### Coefficient of coordinated development

The relative lag between low-carbon development and socio-economic development was analyzed by using the coefficient of coordinated development (shown in Eq. 11).


(11) $$\begin{array}{l} U\;=\;\frac{F\left(x\right)}{G\left(y\right)} \end{array}$$

Where, *U* is the coefficient of coordinated development.

#### Criteria for evaluation

Low-carbon development and socio-economic development should be fully taken into account when the evaluation standard of the coupling development of the two systems was formulated (26). In order to reflect the coupling relationship and development level of the two systems more accurately, the evaluation criteria and basic types of the coupling degree of low-carbon development and socio-economic development on the basis of the coupling coordination level were put in Tables 2–4.

**Table 2:** Criteria for the coupling level between low-carbon development and socio-economic development.

**Level of coupling C**	**Coupling type**
C = 0	Subsystem independent and disordered development
0 < C ≤ 0.3	Low-level coupling
0. <3 < C ≤ 0.5	Rivalry
0.5 < C ≤ 0.8	Running in
0.8 < C <1	High-level coupling
C = 1	Subsystem good resonance coupling and orderly development

**Table 3:** Evaluation standard of coupling coordination level between low- carbon and socio-economic development.

**Coupling coordination level D**	**Coupling coordination state**	**Coupling coordination interval**
0 < D ≤ 0.1	Disorders of recession	Extreme disorder depression
0.1 < D ≤ 0.2		Seriously dysfunctional recession
0.2 < D ≤ 0.3		Moderate disorder decline
0.3 < D ≤ 0.4	Excessive harmonic	Mild dyslexia
0.4 < D ≤ 0.5		Borderline disorder decline
0.5 < D ≤ 0.6		Forced coupling coordination
0.6 < D ≤ 0.7	Low-grade coordination	Primary coupling coordination
0.7 < D ≤ 0.8		Intermediate coupling coordination
0.8 < D ≤ 0.9	High coordination	Good coupling coordination
0.9 < D ≤ 1.0		High quality coupling coordination


**Table 4:** Criteria for evaluating the coefficient of coordinated development between low-carbon and socio-economic development.

**Coefficient of coordinated development U**	**Type of coordinated development**
0.1 < U ≤ 0.8	Low- carbon development lag type
0.8 < U ≤ 1.2	Synchronous low-carbon economy
U>1.2	Socio-economic development lag type

### Synergy degree model

#### Indicator order degree model

Order degree represents the order of the system composition (27). It was calculated by Eq. 12 according to the servitude principle of the synergy theory.


(12) σj(hji) = {hji-min hjimax hji-min hji,i∈(1,k)max hji-hjimax hji-min hji,i∈(k+1,n)

Where, σ_*j*_(*h*_*ji*_) represents the order degree value of each indicator in the subsystem in different years, and σ_*j*_(*h*_*ji*_) ∈ [0, 1], whose value reflects the order degree of the system.

#### System order degree model

Combined with Eq. 12, the order degree measurement indicator of subsystem can be integrated through geometric average (shown in Eq. 13).


(13) $$\begin{array}{l} \mu_{j}\left(h_{j}\right)\;=\;\sqrt[{n}]{\overset{n}{\underset{i=1}{\prod }}\mu_{j}\left(h_{ji}\right)} \end{array}$$

#### System synergetic model

Combined with Eq. 13, the synergy degree of the system can be integrated by Eq. 14 through geometric average and time dimension.


(14) $$\begin{array}{l} cor\left(S_{1},S_{2}\right)\;=\;\varpi \sqrt[{n}]{\overset{n}{\underset{j=1}{\prod }}{\mu_{j}}^{1}\left(h_{j}\right)-{\mu_{j}}^{0}\left(h_{j}\right)} \end{array}$$

Where, $\varpi =\frac{\min\left[{\mu_{j}}^{1}\left(h_{j}\right)-{\mu_{j}}^{0}\left(h_{j}\right)\right]}{\left|\min\left[{\mu_{j}}^{1}\left(h_{j}\right)-{\mu_{j}}^{0}\left(h_{j}\right)\right]\right|}$ represents the action direction of subsystem on the synergy degree of composite system, and the value is −1 or 1. *cor*(*S*_1_, *S*_2_) represents the synergy degree of composite system, and its value range is [−1,1]. Its value is positively correlated with the degree of synergy.

#### Criteria for evaluation

In order to reasonably and effectively reflect the synergistic relationship between low-carbon development and socio-economic development (28), the following evaluation criteria and types based on the core concept of synergism were proposed in Table 5.

**Table 5:** Criteria for evaluating the synergetic degree of low-carbon development and socio-economic development.

**Synergy degree of interval**	**Synergy degree**
[−1, −0.666]	Highly insynergy
[−0.666, −0.333]	Mediumly insynergy
[−0.333, 0]	Mildly insynergy
[0, 0.333]	Mildly synergy
[0.333, 0.666]	Mediumly synergy
[0.666, 1]	Highly synergy

### GM(1,1) model

Gray forecasting method which is short for GM(1,1) later, is a method to predict the gray system (29). For a certain system, it can be divided into white system, gray system and black system according to its internal characteristics which was marked as all known, part known and nothing respectively. The principle of gray forecasting method was used to generate data series with strong regularity on original data through association analysis, and then establish the corresponding differential equation model to predict the future development trend of things as follows:

(1) Define the raw data sequence as follow: $X^{\left(0\right)}=\left\{{x^{\left(0\right)}}_{\left(1\right),}{x^{\left(0\right)}}_{\left(2\right),}\cdots x^{\left(0\right)}\left(n\right)\right\}$, where *x*^(0)^(*k*) ≥ 0, *k* = 1, 2, ⋯, *n*◦
(2) Through the original data sequence, r times of cumulative generation sequence were further obtained by Eq. 15 (one-accumulating generation operator, 1-AGO).

(15) $$\begin{array}{l} x^{\left(r\right)}\left(k\right)=\overset{k}{\underset{i=1}{\sum }}x^{\left(r-1\right)}\left(i\right),k=1,2,\cdots \;,n,r\geq 1 \end{array}$$

(3) The adjacent value was obtained by combining with Eq. 15 to generate sequence (shown in Eq. 16).

(16) $$\begin{array}{l} z^{\left(1\right)}\left(k\right)=\alpha x^{\left(1\right)}\left(k\right)+\left(1-\alpha \right)x^{\left(1\right)}\left(k-1\right),k=1,2,\cdots \;,n \end{array}$$

For convenience, α = 0.5, which was called equal weight neighborhood generating number.

(4) Define the gray differential equation model of GM (1,1) by Eq. 17.

(17) $$\begin{array}{l} d\left(k\right)+az^{\left(1\right)}\left(k\right)\;=\;b \end{array}$$

Where, *d*(*k*) is the gray derivative, *a* is the development coefficient, *z*^(1)^(*k*) is the whitening background value, and *b* is the gray action.

(5) Constructing data matrix *U*, *B*, *Y*, and *Y* = *BU* (shown in Eq. 18).

(18) $$\begin{array}{l} U=\left(\begin{array}{c} a\\ b \end{array}\right), \end{array}$$

$B=\left(\begin{array}{cc} -z^{\left(1\right)}\left(2\right) & 1\\ -z^{\left(1\right)}\left(3\right) & 1\\ \vdots & \vdots \\ -z^{\left(1\right)}\left(n\right) & 1 \end{array}\right)$, $Y=\left(\begin{array}{c} x^{\left(0\right)}\left(2\right)\\ x^{\left(0\right)}\left(3\right)\\ \vdots \\ x^{\left(0\right)}\left(n\right) \end{array}\right)$ Where, according to the least square method, it can be obtained by *U* = (*B*^*T*^*B*)^−1^*B*^*T*^*Y*◦

(6) Then, the corresponding function of the time obtained was formed, and the sequence of predicted numbers of the accumulated generation was obtained by IAGO (shown in Eq. 19 and Eq. 20).

(19) $$\begin{array}{l} x^{\left(1\right)}\left(k\right)=\left(x^{\left(0\right)}\left(1\right)-\frac{b}{a}\right)e^{-a\left(k-1\right)}+\frac{b}{a},k=1,2,\\ \;\cdots ,n \end{array}$$

(20) $$\begin{array}{l} x^{\left(0\right)}\left(k\right)\;=\;x^{\left(1\right)}\left(k\right)-x^{\left(1\right)}\left(k-1\right),k=1,2,\cdots \;,n \end{array}$$

(7) Later, residual test was performed on predicted and actual values by Eq. 21.

(21) $$\begin{array}{l} \varepsilon \left(k\right)\;=\;\frac{x^{\left(0\right)}\left(k\right)-{\overset{\wedge }{x}}^{\left(0\right)}\left(k\right)}{x^{\left(0\right)}\left(k\right)},k=1,2,\cdots \;,\;n \end{array}$$

When |ε(*k*)| < 0.1, it is considered that the prediction meets the higher requirement; when |ε(*k*)| < 0.2, it is considered that the prediction meets the general requirement.

### Data collection

The data 2011 to 2020 in Anhui Province (China) was collected from websites such as the National Bureau of Statistics of the Peoples' Republic of China, Anhui Provincial Bureau of Statistics, Anhui Provincial Department of Ecological Environment, and Anhui Provincial Development and Reform Commission.

## Results

### Result analysis of entropy weight method

Through the above data collection, the raw data of each indicator from 2011 to 2020 was obtained and processed as follow:

(1) Combined with Eq. 1 & Eq. 2, the raw data was substituted into the standardization. And then, corresponding standardized values of each indicator were obtained (Table 6).

**Table 6:** The standardized values of each indicator from 2011 to 2020.

**Indicator**	**2011**	**2012**	**2013**	**2014**	**2015**	**2016**	**2017**	**2018**	**2019**	**2020**
X1	0.010	0.363	0.363	0.363	0.363	0.363	0.363	0.628	0.628	1.000
X2	0.998	1.000	0.849	0.718	0.376	0.255	0.010	0.144	0.171	0.544
X3	0.010	0.040	0.229	0.319	0.419	0.530	0.696	0.925	1.000	1.000
X4	0.955	0.746	0.446	0.200	0.010	0.213	0.466	0.815	1.000	1.000
X5	1.000	0.928	0.509	0.456	0.506	0.481	0.326	0.069	0.058	0.010
X6	0.010	0.086	0.156	0.421	0.516	0.602	0.739	0.819	1.000	0.993
X7	0.010	0.115	0.216	0.298	0.399	0.510	0.625	0.760	0.909	1.000
X8	0.010	0.026	0.047	0.136	0.267	0.461	0.487	0.675	0.974	1.000
X9	0.010	0.085	0.165	0.260	0.308	0.401	0.542	0.670	0.808	1.000
X10	0.010	0.126	0.229	0.325	0.421	0.532	0.643	0.731	0.814	1.000
X11	0.010	0.046	0.118	0.237	0.392	0.598	0.729	0.833	0.954	1.000
X12	0.010	0.057	0.107	0.110	0.200	0.231	0.376	0.512	0.667	1.000

In Table 6, the measurement units of various indicators are not unified, they should be standardized before calculating the comprehensive development level with them. Then, the absolute value of indicators can be converted into relative values, so as to solve the homogenization problem of different quality indicators.

(2) Combined with Eq. 3 & Eq. 4, the standardized values were substituted to obtain the information entropy of each indicator (Table 7).
(3) Combined with Eq. 5, the comprehensive weight of each indicator in the evaluation system was obtained (Table 7). Meanwhile, the weight represents the importance of the studied indicator to the studied system.

**Table 7:** Information entropy and weight distribution of each indicator.

**Indicator**	**X1**	**X2**	**X3**	**X4**	**X5**	**X6**	**X7**	**X8**	**X9**	**X10**	**X11**	**X12**
Information entropy(ej)	0.925	0.881	0.873	0.904	0.861	0.886	0.890	0.814	0.874	0.899	0.858	0.824
Weight (wj)	0.050	0.079	0.084	0.064	0.092	0.075	0.073	0.123	0.083	0.067	0.094	0.116

In Table 7, information entropy represents the variation degree of indicators. According to the principle of information entropy, the smaller the value is, the greater the variation degree of indicators is and the more information it provides, which plays a relatively larger role in the comprehensive evaluation. Then, the larger the weight proportion involved in the following steps is.

(4) Combined with Eq. 6, Eq. 7, & Eq. 8, the index of low-carbon development and socio-economic development can be calculated. After obtaining standardized values and comprehensive weights of all indicators through the above steps, the low-carbon development index can be obtained by Eq. 6. Meanwhile, the socio-economic development index can be obtained by Eq. 7. Furthermore, the comprehensive development index can be obtained by Eq. 8.

**Figure 2:**
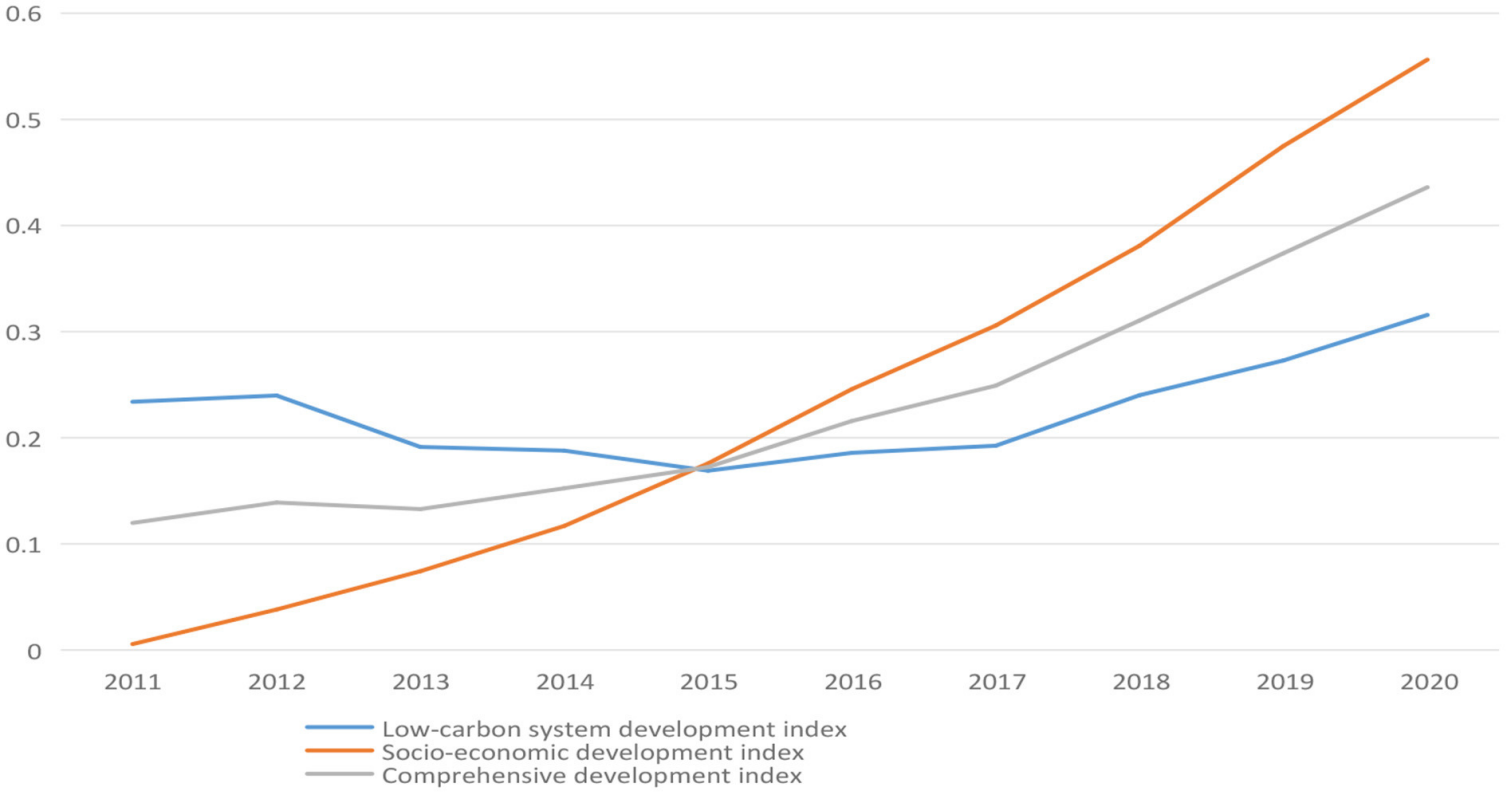
Change trend in Anhui province.

And then, according to the above three indexes in Anhui province from 2011 to 2020, the corresponding trend changes were obtained (Figure 2).

In Figure 2, although the comprehensive indicator of low-carbon development and socio-economic development in Anhui province fluctuated in a small range from 2012 to 2013, it showed an overall upward trend. Later, the indicator of low-carbon development showed a downward trend from 2011 to 2015, and then rose slowly from 2015 to 2020. The low-carbon development index in China from 2010 to 2018 showed a trend of annual growth except for a slight decrease in 2011 compared with 2010.

According to the above analysis, it is found that the total carbon emissions and fossil energy consumption of the studied region increased significantly from 2011 to 2014. However, with the release of the National Climate Change Plan (2014–2020), all regions in China began to complete the low-carbon development construction as the target orientation. As a result, the growth rate of total carbon emissions in Anhui province and even the whole country slowed down significantly in 2015. Furthermore, the trend of low-carbon development has been improved to some extent. Meanwhile, the convening of the 19th National Congress of the Communist Party of China in 2017 emphasized the importance of building a modern green economic system and taking the road of high-quality socio-economic development, which is one of the necessary conditions for a more stable trend of low-carbon development (30).

### Result analysis of coupling coordination model

Coupling coordination model analysis in Anhui province was processed as follow:

(1) Combined with Eq. 9, the coupling level (*C*) can be calculated in combination with low-carbon development index (*F(x)*), socio-economic development index *(G(y))* and comprehensive development index (*T*) of Anhui province in 2011-2020 obtained from the previous section.
(2) Combined with Eq. 10, the coupling coordination level (*D*) can be calculated, as well as comprehensive development index (*T*) and coupling level (*C*).
(3) Combined with Eq. 11, the coordinated development index (*U*) can be calculated, as well as the low-carbon development index (*F(x)*) and the socio-economic development index (*G(y)*).

Then, according to Tables 2–4, the results including the types of the coupling degree (*C*), the coupling coordination level (*D*) and the coordinated development index (*U*) in Anhui province from 2011 to 2020 can be obtained (Table 8).

**Table 8:** Coupling degree of low-carbon development in Anhui province.

**Year**	**C**	**Coupling type**	**D**	**Coupled state**	**U**	**Type of coordinated development**
2011	0.001	Low-level coupling	0.009	Extreme disorder depression	42.047	Economic development lag type
2012	0.106	Low-level coupling	0.121	Seriously dysfunctional recession	6.295	Economic development lag type
2013	0.523	Running in	0.263	Moderate disorder decline	2.577	Economic development lag type
2014	0.846	High-level coupling	0.359	Mild dyslexia	1.608	Economic development lag type
2015	0.999	High-level coupling	0.415	Borderline disorder decline	0.961	Synchronous low-carbon economy
2016	0.943	High-level coupling	0.451	Borderline disorder decline	0.756	Low-carbon development lag type
2017	0.853	High-level coupling	0.461	Borderline disorder decline	0.630	Low-carbon development lag type
2018	0.853	High-level coupling	0.515	Forced coupling coordination	0.630	Low-carbon development lag type
2019	0.796	Running in	0.546	Forced coupling coordination	0.574	Low-carbon development lag type
2020	0.789	Running in	0.586	Forced coupling coordination	0.568	Low-carbon development lag type

In Table 8, the coupling coordination level between low-carbon development and socio-economic development in Anhui province generally showed a stable and positive trend. Furthermore, 2019 years ago, the coupling level between low-carbon development and socio-economic development was from low to high, and the coupling coordination level was also from extreme imbalance recession to force coupling coordination. It means that the coordination type was from socio-economic development lag to synchronous development. Later, it was transformed into the lag state of low-carbon development.

The reasons for the above phenomenon are still closely related to the strategic advantages of Anhui Province in the past few years, which attached importance to the improvement of socio-economic benefits and actively built the high-quality development of strategic emerging industries. Furthermore, low-carbon development and socio-economic development were more and more harmonious, but socio-economic in Anhui province was still a type of huge energy consumption, so it is inevitably that low-carbon development lagged behind. Therefore, the socio-economic development is necessary and also has a certain role to promote the coordinated development in the future. It will certainly promote the synchronous realization of low-carbon development. Additionally, from 2019 to 2020, the coupling type of the two systems decreased to run-in, which also proved that the unbalanced and inadequate development of Anhui province was still prominent. Therefore, low-carbon development was facing new opportunities and challenges. In this situation, it is more necessary to maintain the strategic of low-carbon development.

### Results analysis of synergy degree model

In this section, the elements of the above indicator system (Table 1) were applied according to the raw data collected in Anhui province as follow:

(1) Combined with Eq. 12, the raw data was standardized (Table 6), which has been obtained above.
(2) Combined with Eq. 13, the μ_*j*_(*h*_*j*_) combination of the four criteria layers was calculated according to the indexes covered by the different criteria layers and their normalized values. Then, combined with Eq. 13 again, the μ_*j*_(*h*_*j*_) combination of target layers *S1* and *S2* was calculated according to the criteria layer values covered by different target layers. The results were shown in Table 9.
(3) Combined with Eq. 14, the synergy degree of target Layer *S1* and *S2* was obtained, and the changes of synergy degree in Anhui province from 2012 to 2020 were shown in Figure 2.

**Table 9:** The order degree of criteria layer and target layer in Anhui province from 2011 to 2020.

	**2011**	**2012**	**2013**	**2014**	**2015**	**2016**	**2017**	**2018**	**2019**	**2020**
Resource environment endowment	0.046	0.243	0.413	0.436	0.385	0.366	0.136	0.438	0.475	0.816
Energy environment endowment	0.212	0.391	0.328	0.337	0.138	0.395	0.482	0.359	0.386	0.215
Economic development endowment	0.010	0.064	0.119	0.219	0.320	0.455	0.548	0.701	0.894	1.000
Social development endowment	0.010	0.069	0.143	0.204	0.321	0.419	0.561	0.678	0.803	1.000
Low-carbon system (S1)	0.099	0.308	0.368	0.383	0.230	0.380	0.256	0.396	0.428	0.419
Socio-economic system (S2)	0.010	0.066	0.130	0.211	0.320	0.437	0.554	0.689	0.847	1.000

**Figure 3:**
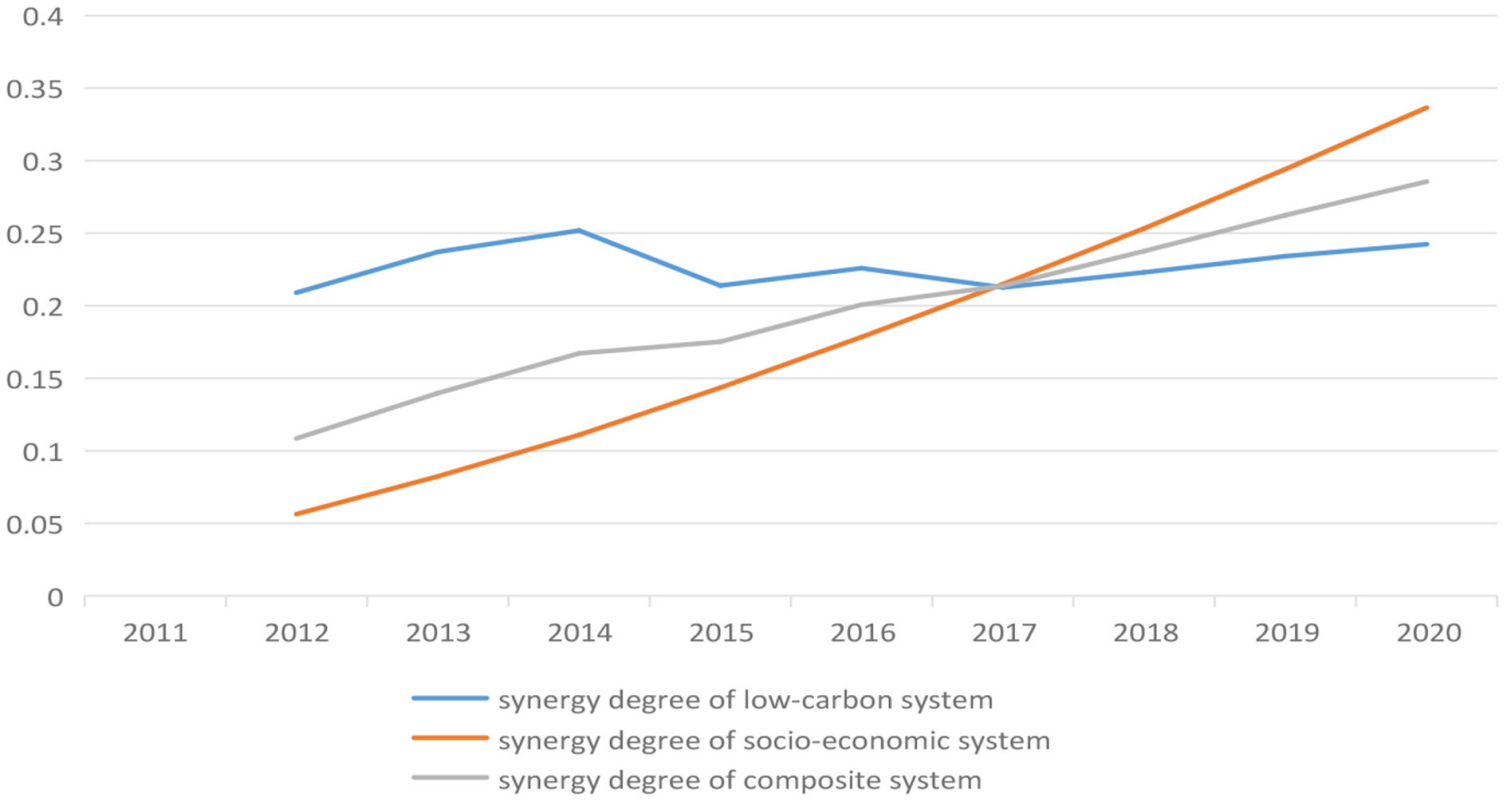
The trend of synergy degree between low-carbon development and socio-economic development in Anhui province.

According to Tables 5, 9, and Figure 3, it can be found that in the context of low-carbon development and socio-economic development, except for 2015 and 2017, the level of two systems on the synergy degree was in progress from mild synergy to moderate synergy. Furthermore, synergy degree rise steadily, but its gap was narrowing. It means that two systems were gradually from chaos to order the stability of the structure.

The above result showed in the process of low-carbon development in the 21st century means that great efforts have been made in Anhui province. But it still was necessary to improve the energy efficiency, vigorously develop the clean energy and actively promote the optimization and upgrading of industry. This is because that low-carbon development can be a momentum of socio-economic development, and socio-economic development can also promote the reduction of carbon emissions. If these measures can be pushed forward consistently, a stable synergistic structure of their interaction will be eventually spontaneously achieved in the future.

### Results analysis of gray forecasting method

**Figure 4:**
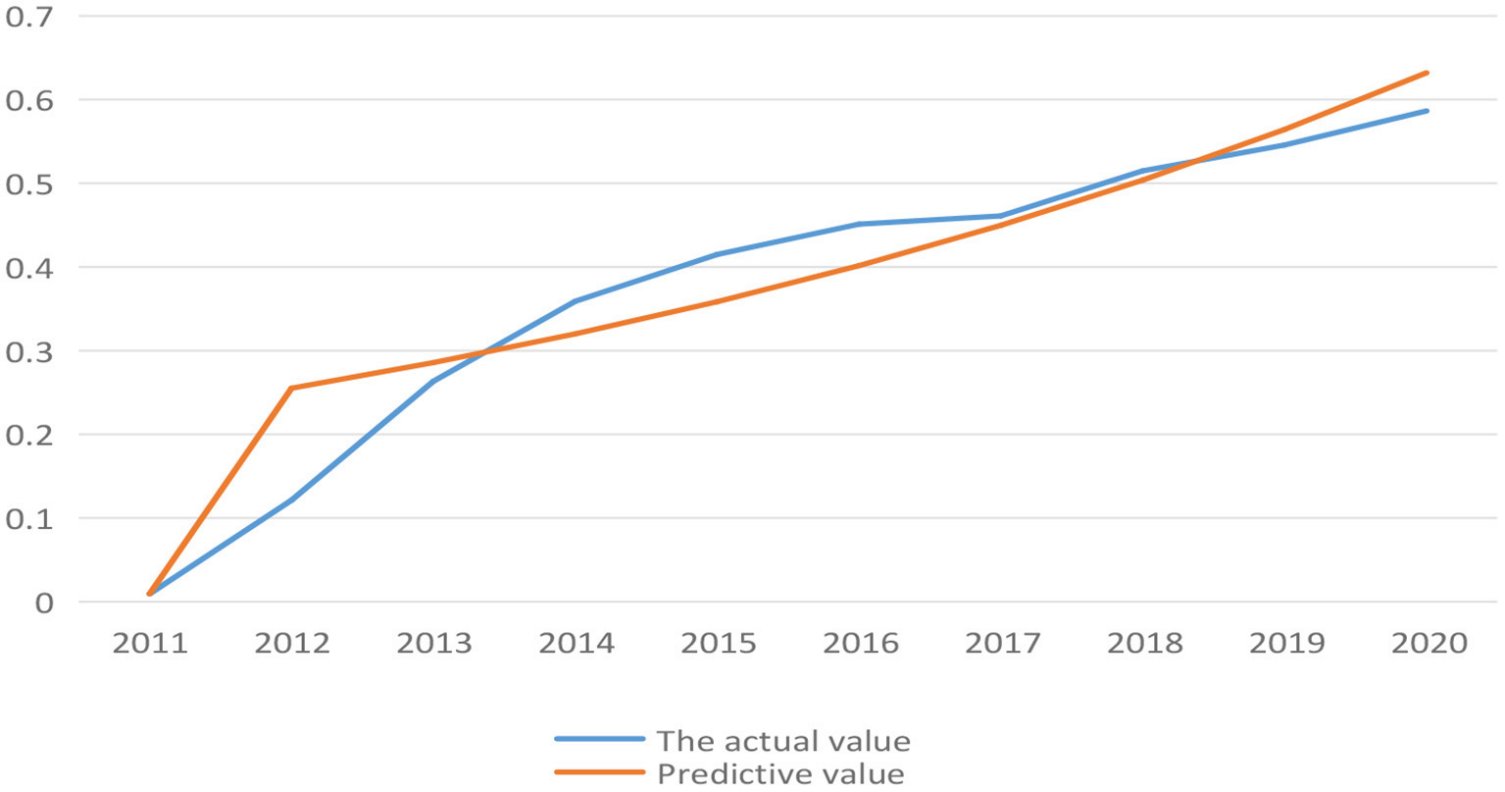
Comparison between actual value and predicted value of coupling coordination level of low-carbon development during 2011-2020.

**Figure 5:**
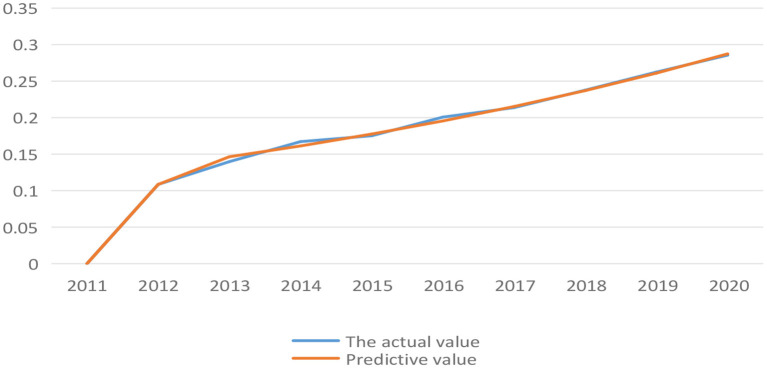
Comparison between actual value and predicted value of synergy degree of low-carbon development in Anhui province during 2012-2020.

One of the necessary conditions for forecasting the trend is that the error between the predicted value and the actual value is small. In this section, it is necessary to test it when the gray forecasting method was used in Anhui province as follow:

(1) Combining the data analysis part above, the initial value of coupling coordination degree and synergy degree *X*^(0)^ was obtained (Table 10).
(2) Combined with Eq. 15–21, the data is processed by excel, the predicted value of coupling coordination level from 2011 to 2020 and the predicted value of synergy degree from 2012 to 2020 were compared with the actual value (Figures 4, 5).

**Table 10:** Initial value of coupling coordination level and synergy degree.

**X(0)**	**2011**	**2012**	**2013**	**2014**	**2015**	**2016**	**2017**	**2018**	**2019**	**2020**
Coupling coordination level	0.009	0.121	0.263	0.359	0.415	0.451	0.461	0.515	0.546	0.586
Synergy degree	-	0.109	0.140	0.167	0.175	0.201	0.214	0.238	0.262	0.286

In Figures 4, 5, it can find that there is a large gap between the predicted value and the actual value of coupling coordination level before 2017. But as time goes by, the prediction became more accurate, and the predicted value of synergy degree was highly consistent with the actual value. It was reasonable to use gray forecasting model to predict the coupling coordination level and synergy degree between low-carbon development and socio-economic development in Anhui province.

(3) Combined with Eq. 15–21 again, the specific value and residual error also can be presented synthetically based above data (Table 11).

**Table 11:** Residual changes of coupling coordination level and synergy degree of low-carbon development in Anhui province from 2011 to 2020.

**Year**	**Actual coupling coordination level**	**Predict coupling coordination level**	**Residual**	**Actual synergy degree**	**Predictive synergy degree**	**Residual**
2011	0.0095	0.0095	0	-	-	-
2012	0.1213	0.2551	−1.1026	0.1085	0.1085	0
2013	0.2634	0.2857	−0.0846	0.1397	0.1464	−0.0480
2014	0.3590	0.3200	0.1085	0.1671	0.1612	0.0354
2015	0.4149	0.3585	0.1361	0.1752	0.1775	−0.0134
2016	0.4511	0.4015	0.1100	0.2007	0.1955	0.0262
2017	0.4609	0.4497	0.0244	0.2137	0.2152	−0.0071
2018	0.5148	0.5037	0.0216	0.2377	0.2370	0.0027
2019	0.5457	0.5642	−0.0339	0.2624	0.2610	0.0055
2020	0.5864	0.6319	−0.0777	0.2856	0.2873	−0.0061

In Table 11, it can be found that the absolute value of coupling coordination residuals is less than 0.2 except for 2012, indicating that the prediction results are reasonable. Among them, 2014-2016 was a general fit, while 2013 and 2017-2020 were highly fit. Furthermore, the synergistic residuals were all in a highly fit, and the residual comparison analysis further proved that the GM(1,1) can be used to predict coupling coordination level and synergy degree from 2022 to 2030 in Anhui province (shown in Table 8).

(4) Combined with Eq. 15–21 again, the value of coupling coordination level and synergy degree of Anhui province in 2020 was taken as the initial value and analyzed by the same program. And then, the data of coupling coordination level and synergy degree of Anhui Province in 2022-2030 are predicted (Table 12).

**Table 12:** Prediction results of coupling coordination level and synergy degree of low-carbon development in Anhui province from 2022 to 2030.

**Year**	**Coupling coordination predicted value**	**Synergy degree predicted value**
2022	0.7928	0.4948
2023	0.8880	0.5448
2024	0.9946	0.5999
2025	1.1140	0.6606
2026	1.2477	0.7274
2027	1.3976	0.8009
2028	1.5654	0.8819
2029	1.7533	0.9711
2030	1.9638	1.1778

In Table 12, both the predicted values of coupling coordination level and synergy degree in Anhui province shows an upward trend. The coupling coordination level will enter the type of high-quality coupling coordination in 2022, and reach the optimal level and continue in 2024. The synergy degree will enter the highly synergistic state in 2025, and reach the optimal degree and continue in 2029. By comparing the two, it can be found that the realization of the high quality level of coupling coordination is scientific and reasonable before entering the high level of coordination. Therefore, coordination of this balance state is the premise for low-carbon development and socio-economic development in Anhui province to move toward an orderly structure and maintain dynamic stability. Furthermore, the above results also prove that the feasibility to realize the dual carbon goal before 2030 under the premise of realizing highly coordinated low-carbon development in Anhui province.

## Discussion

The emergence of low-carbon economy is directly motivated by the United Nations Framework Convention on Climate Change (CONVENTION) and Kyoto Protocol (PROTOCOL) reached in 1992 and 1997 (31). The CONVENTION and PROTOCOL provide an important institutional foundation for global low-carbon development. However, there are two problems as follow: The first is the responsibility problem. Low-carbon economy in the CONVENTION and PROTOCOL arbitrarily turns carbon emissions into a public good and make low-carbon development a mandatory responsibility. However, this concept of compulsion is in conflict with the dominant concept of development centralism worldwide (32). In 2015, although the Paris Agreement made a major adjustment to the “Kyoto Model” and added the responsibility model of “voluntary emission reduction”, the responsibility problem of low-carbon economy is still not well solved, and the necessity of low-carbon development in developing countries needs to be scientifically evaluated (33, 34). If the conditions of low-carbon development and socio-economic development can be identified, then the responsibility problem of low-carbon economy will be easier to be implemented. The second is the implementation problem. Low-carbon economy in the CONVENTION and PROTOCOL simply exempts developing countries from their obligations of carbon emission reduction, which is resisted by almost all developed countries except US and substantially weakens the implementation of low-carbon development (35). The Paris Agreement calls for “differentiated responsibility” and global “shared responsibility” to replace the principle of “southern impunity”. However, the implementation of low-carbon economy still needs to be further accurately predicted in developing countries in order to fit their actual situation. Therefore, accurate identification of the dynamic relationship between low-carbon development and socio-economic development is more conducive to the development of low-carbon economy.

In terms of low-carbon economy in Anhui Province, which is a province with relatively active economic development has the potential for implementation to realize low carbon development, such as high technological level, strong development momentum, easy access to investment projects, and easy access to developed countries or regions of technical and financial assistance. Meanwhile, developed countries will actively implement technology-guided and market-driven strategies through international cooperation in order to achieve carbon emission reduction targets, so as to promote the technological progress of low-carbon development. This can provide operational capital and operable clean energy technology for carbon emission reduction in Anhui Province. Furthermore, developed countries choose partners with no or relatively small emission reduction responsibilities to provide financial assistance or technology transfer, which can also help partners including Anhui province with low cost emission reduction. Moreover, the CONVENTION and PROTOCOL also sets aside certain space for provinces in developing countries on the principle of “common but differentiated responsibilities” for carbon emissions, which is conducive to the development of low-carbon economy in Anhui Province. After all, the implementation of carbon emissions in Anhui Province is not very long, and it is still in the primary development stage, so there is much room for the improvement of carbon emission reduction. Additionally, as a province in a developing country, the cost of carbon emission reduction in Anhui province is relatively low. Combined with the advantages of relatively cheap labor cost and huge market size, Anhui province has the advantage of setting up new enterprises, using new technology and new equipment, as well as the cost advantage of upgrading old enterprises and old equipment, so it is feasible to realize the develop low-carbon economy with low cost in Anhui province.

Combined with the research results of this paper and the focus of academic circle on low-carbon economy, the following discussion points were formed.

### Low-carbon development lagged behind socio-economic development

In this paper, a comprehensive indicator evaluation system was analyzed *via* coupling coordination level model and synergy degree model to verify that the current conditions of low-carbon development and socio-economic development in Anhui province. It was found that low-carbon development lagged behind socio-economic development. Relevant studies also confirm that there is a certain difference between urban low-carbon development index and socio-economic development index in China, in which socio-economic development index are generally higher than that of low-carbon development index (36). And countries with faster socio-economic development have a significant positive effect on their per capita CO^2^ emissions (37). For example, with the rapid socio-economic development, the increase of resources and energy consumption leads to the gradual increase of carbon emissions, then the low-carbon development index also gradually decreases. Later, with the transformation of socio-economic development from high-speed to high-quality, the advocacy of low-carbon life gradually increases, and the advocacy of low-carbon development index also gradually increases, but it still lags behind the speed of steady improvement of socio-economic development index. In order to promote the process of low-carbon development, it is extremely necessary to control the level of carbon emissions, promote low-carbon development in the energy sector and strengthen the construction level of low-carbon development policies.

### Low-carbon development and socio-economic were mutually beneficial, but their coupling coordination is relatively weak

Through the study above, it was found that the coupling coordination level of low-carbon development and socio-economic development is in the stage of barely coupling in Anhui province. However, the coordination between low-carbon development index and socio-economic development index of different types of cities has been improving, and the coordination difference has converged on the whole. Meanwhile, although low-carbon development lags behind socio-economic development, the two have been actively coupled (38). Not only that, low-carbon development benefits and socio-economic development benefits can complement each other, and the realization of environmentally friendly socio-economic development is one of the goals to achieve high-quality low-carbon development. Furthermore, based on the actual situation, it can be found that great efforts have been made to promote low-carbon development in Anhui Province. While promoting low-carbon development and socio-economic development, it is committed to focusing on improving energy efficiency, vigorously developing clean energy, and actively promoting industrial optimization and upgrading. Moreover, the comprehensive research content of this paper can prove that low-carbon development can become the driving force of socio-economic development, and socio-economic development can also promote the reduction of carbon emissions. Additionally, the GM(1,1) model was used to predict the coupling coordination level from 2011 to 2020, and it is found that the predicted data has a high consistency with the actual data. This model can be used to further predict coupling coordination level and synergy degree from 2022 to 2030. After the two systems has gone through the stage of “antagonism → running-in → coordination”, as long as the low-carbon development path continues to be adhered, high-quality coupling coordination will inevitably be spontaneously formed to promote the formation of stable collaborative structure (39).

### The coordinated level of low-carbon development and socio-economic development were continuously improved, but the realization of their ordered structure is quite difficult

Combined with the research, the coordination level of low-carbon development and socio-economic development is in a good situation. For example, the overall development level of low-carbon economy and high-quality economy in China is on the rise, and it is currently in the coordination stage. Furthermore, the factors of the two systems are intertwined and interacted with each other, making it gradually transform the original disorderly system form into a highly coordinated and orderly system. It is one of the important channels to achieve orderly structure to construct regional collaborative low-carbon development paths from industrial structure optimization, energy structure transformation and low-carbon technological innovation (16, 40). For example, it was found that from 2019 to 2020, the coupling type of the two systems drops to the run in period, which also indicates that the unbalanced and inadequate development of Anhui province is still prominent, and low-carbon development is facing new opportunities and challenges. Additionally, combined with gray forecasting model, it can find that the coupling coordination level will enter the high-quality coupling coordination type in 2022, the coordination level will enter the high coordination state in 2025, and the coupling coordination level will enter the plateau stage in 2024 and 2029 respectively. These studies confirm that low-carbon development and socio-economic development are bound to travel a bumpy road.

## Conclusions

Through current situation analysis and trend prediction, some conclusions are formed as follow:

### Highlighting the strategic guidance of sustainable development

Under the new situation, carbon emission reduction is still outstanding (41). In the future, with sustainable development as the guide, relying on technological innovation, system optimization, industrial upgrading, clean energy development and so on, a series of feasible measures in Anhui province should be used to achieve the low-carbon development of the energy flow and the flow of resources recycling to reduce the high carbon energy consumption and reduce carbon emissions as follow: First, it is necessary to clarify the strategic layout and realistic requirements of the “dual carbon” strategy. Specifically, it is necessary to guide the public to understand the country's “dual carbon” strategy, strengthen the awareness of low-carbon development, and practice the concept of green life. Second, it is unavoidable to focus on the “dual carbon” strategy and complete low-carbon development plans. This is because that with the acceleration of urbanization, low-carbon development has become an important indicator of high-quality socio-economic development. Therefore, a development model with low energy consumption, low environment pollution and low carbon emissions should be actively explored to create a new development pattern. Finally, it is the right thing to implement the new development concept. The total energy consumption in Anhui province is large and accounts for a high proportion. Therefore, it is necessary to use “carbon peak” to force “carbon emission reduction”, increase the proportion of non-fossil energy use, gradually expand the supply of clean energy, realize energy transformation and empower. And then, low-carbon development can be realized earlier.

### Perfecting new regulatory mechanisms for carbon emission reduction

Carbon emission reduction have a comprehensive decision deployment, so it is necessary to promotes relevant actions on the road of carbon emission reduction, as well as insisting on pioneering and innovation as follow: first, the understanding of the laws of social governance against the background of the “dual carbon” strategy should be deepened. And then, more social and market entities can be encouraged to participate in social governance to improve the effectiveness of social governance in a more diverse way, and share the fruits of social governance more equitably. Second, a new pattern of people-centered, low-carbon governance needs to build with the help of block chain, big data, artificial intelligence and other modern technologies. And then, the socialized and intelligent governance model can be innovated to realize the efficient governance of carbon emission reduction. Finally, it is the right thing to introduce and cultivate high-level innovative and entrepreneurial talents and improve the incentive mechanism for the introduction of outstanding talents. And then, a diversified and flexible modern governance platform can be built to solve key technological problems of carbon emission reduction, and an intelligent support for carbon emission reduction governance can be provided.

### Innovating the science and technology to resolve major development crises

The global low-carbon technology has become more and more mature, but the potential of carbon emission reduction still should be tapped in Anhui province. Therefore, capital and technological advantages should be integrated to obtain the benefits brought by historical carbon emissions (10). First, low-carbon technologies should be promoted under the guidance of a new development philosophy and new technologies to accelerate the optimization and upgrading of industrial structure. Specifically, implementation plans should be formulated and improved in the fields of electricity, construction, transportation and other industries to tackle key issues in low-carbon technologies. Second, the technological innovation of key enterprises should be promoted to reduce dependence on traditional energy sources. Specifically, by exploring the “dual carbon” adaptation model, the path of low-carbon development should be perfected. And then, low-carbon development can be promoted and upgraded to achieve the “dual carbon” target. Finally, the development of artificial carbon conversion technology is necessary to effectively reduce the total carbon emissions of fossil energy. Specifically, on the basis of energy stocks and supported by scientific and technological innovation, the deployment of relevant de-carbonization, zero-carbon and negative emission technologies can be comprehensively strengthened. And then, carbon capture, utilization and storage technologies can be promoted.

## Data availability statement

The original contributions presented in the study are included in the article/supplementary material, further inquiries can be directed to the corresponding author/s.

## Author contributions

QX: conceptualization, literature analyze, and writing—review. ZY: conceptualization, literature search, and writing—review and editing. All authors agreed with the content and all gave explicit consent to submit the manuscript.

## Funding

This work was supported by the following programs: Anhui Provincial Philosophy and Social Science funding project: study on the index system of Rural revitalization of Revolutionary Fellow villagers in Dabie Mountains (No. AHSKQ2021D19).

## Conflict of interest

The authors declare that the research was conducted in the absence of any commercial or financial relationships that could be construed as a potential conflict of interest.

## Publisher's note

All claims expressed in this article are solely those of the authors and do not necessarily represent those of their affiliated organizations, or those of the publisher, the editors and the reviewers. Any product that may be evaluated in this article, or claim that may be made by its manufacturer, is not guaranteed or endorsed by the publisher.
